# Supplementary material for: Histological, Physiological and Transcriptomic Analysis Reveal Gibberellin-Induced Axillary Meristem Formation in Garlic (Allium sativum)
Source: Plants (Basel). 2020 Jul 31;9(8):970. doi: 10.3390/plants9080970 (PMC7464525; doi:10.3390/plants9080970)
Supplement: Supplementary file 1 [file plants-09-00970-s001.pdf]

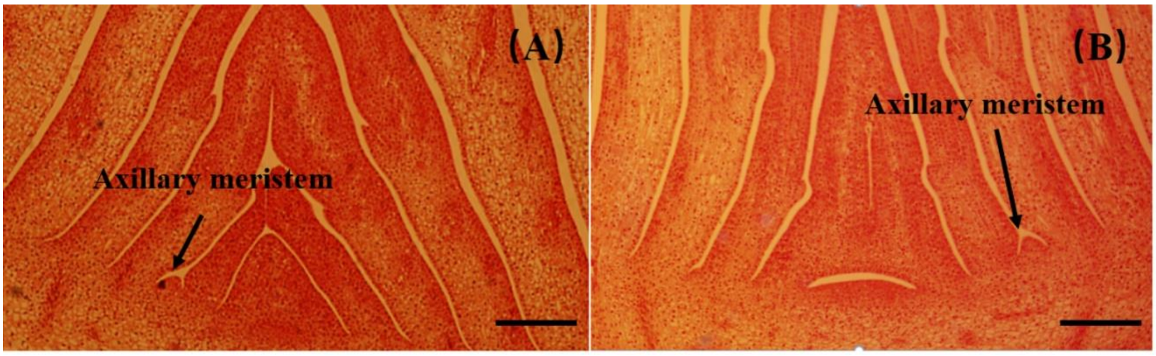

16 days after GA_3 treatment **60 days after GA_3 treatment**

Figure S1 Histological studies on axillary meristem development of GA_3-treated plant. (A), axillary meristem arose at 16 days after GA_3 treatment. (B), axillary meristem arose at 60 days after GA_3 treatment. Scale bars in A and B are 200 μm in length.

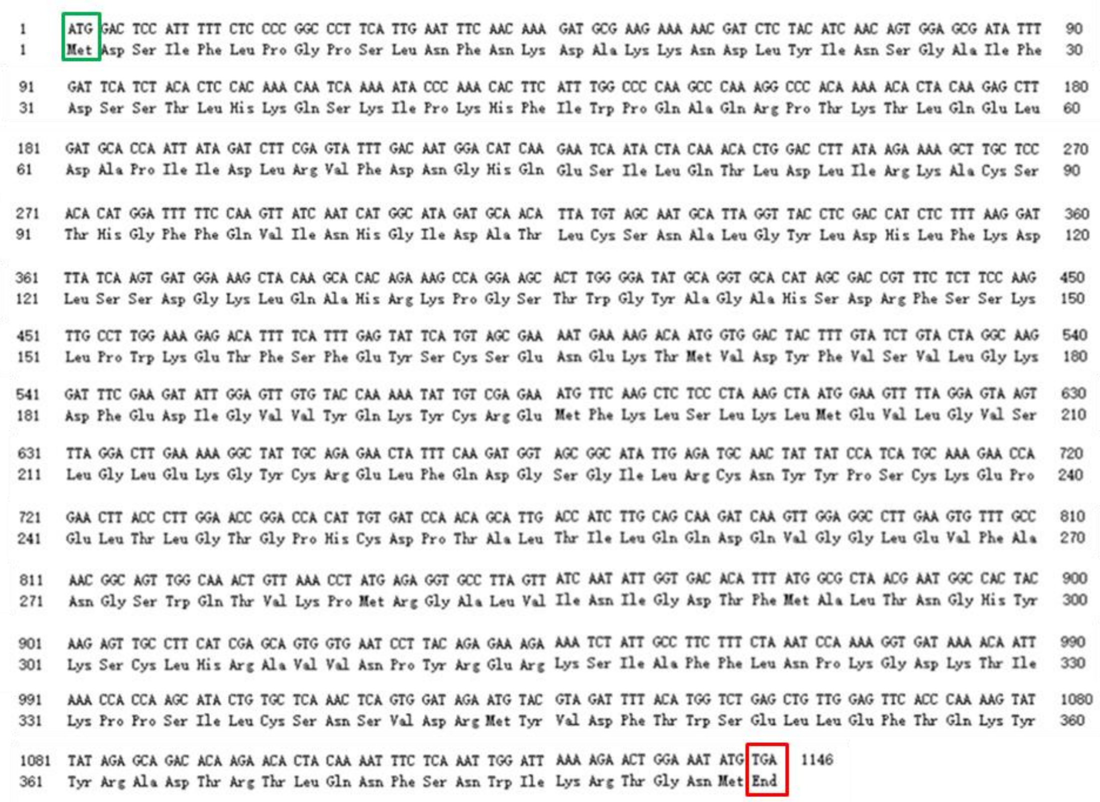

Figure S2 The nucleotide sequence and the deduced amino acid sequence of *AsGA20ox* CDS.

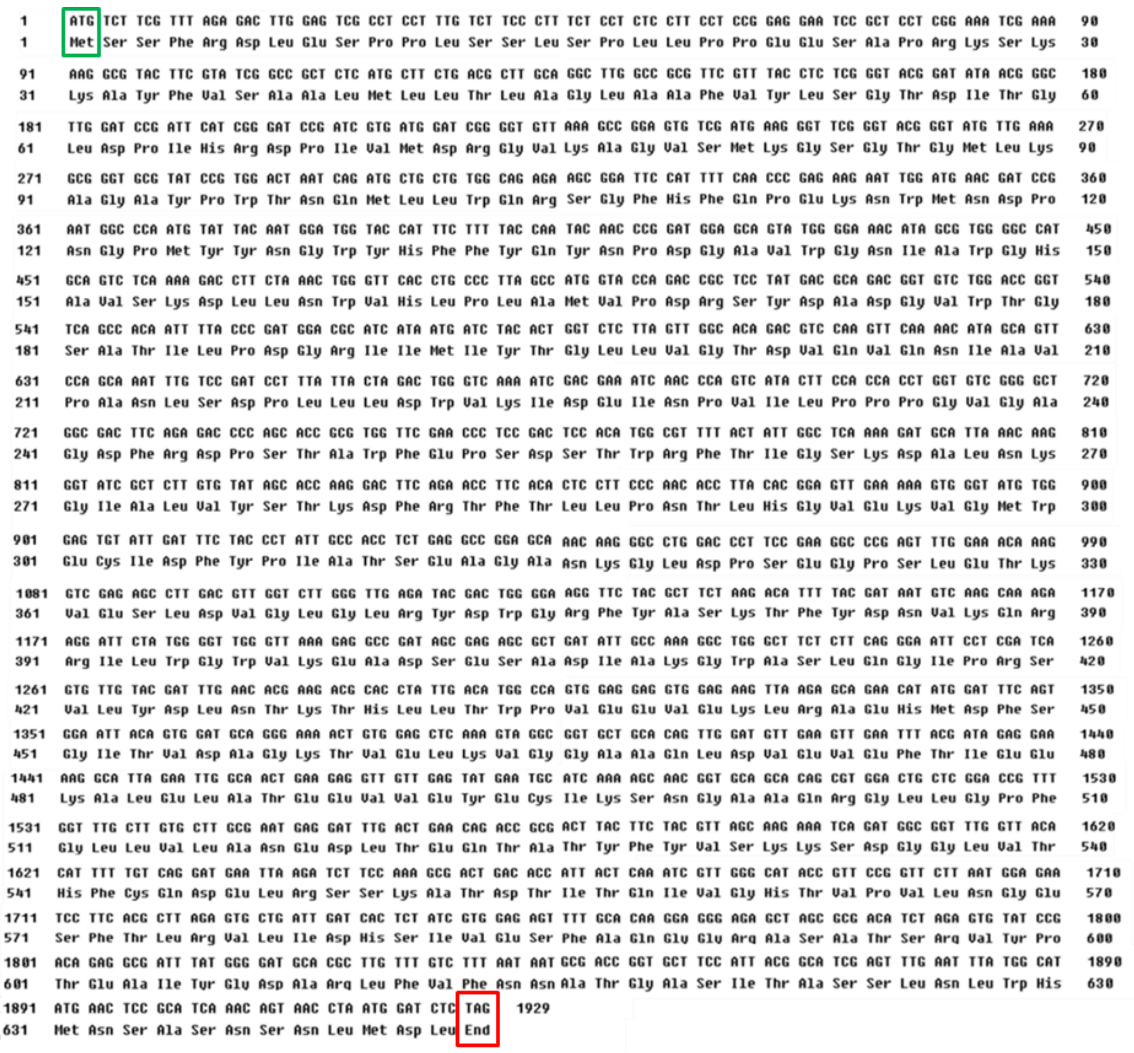

Figure S3 The nucleotide sequence and the deduced amino acid sequence of *AsINV* CDS.

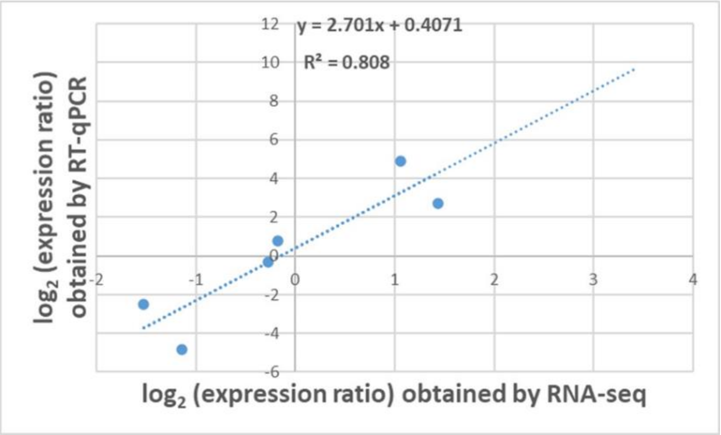

Figure S4 Comparison between the log2 of expression ratios of DGEs obtained from RNA-seq and RT-qPCR.

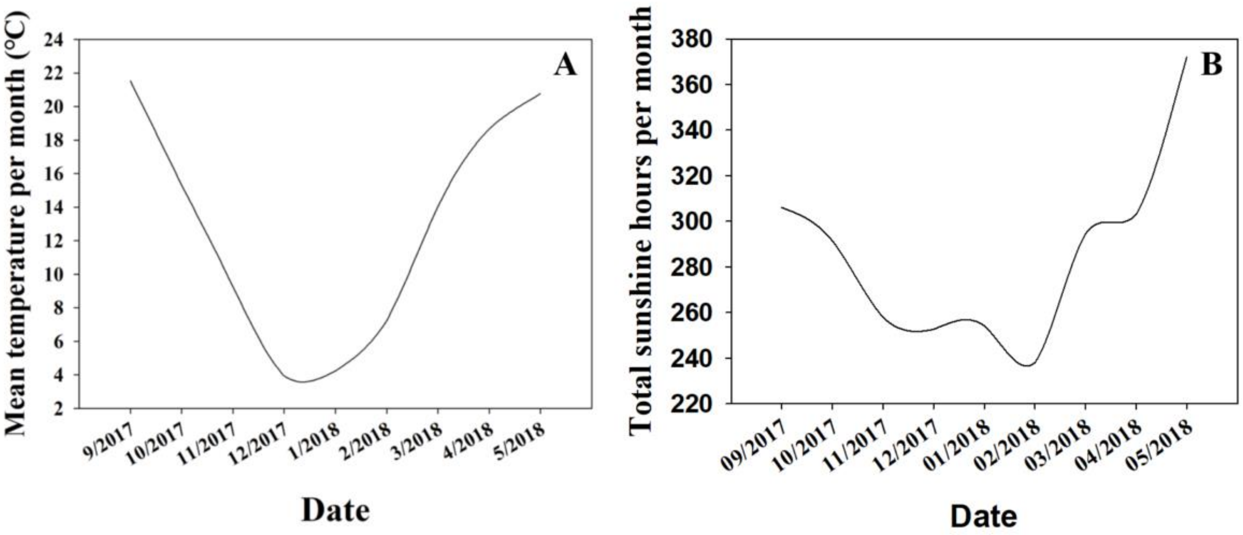

Figure S5 Average temperature per month and total sunshine hours per month in the plastic tunnel. The data show the mean of air temperature within a month (A) and the total hours of illumination intensity higher than 2 klux within a month (B).

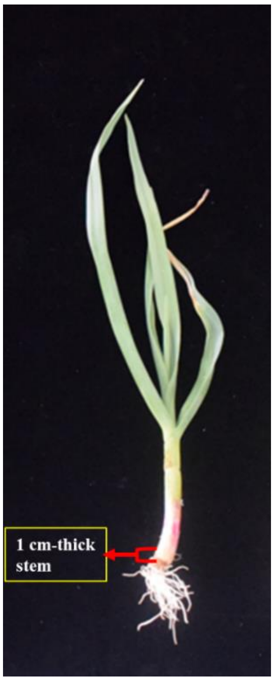

Figure S6 Sample for measuring endogenous plant hormone level, sugars content, soluble protein content and related genes expression in 1 cm-thick stem containing shoot apical meristem.

Table S1 List of 159 DEGs in this study

gene_id	GA_readcount	CK_readcount	Swissprot annotation
Cluster-32430.84552	121.5226452	0	Putative casein kinase II subunit beta-4
Cluster-32430.98519	67.49916236	0	Protein Rf1, mitochondrial
Cluster-66662.0	0.308096439	60.57114749	None
Cluster-32430.176693	0	51.36034364	None
Cluster-32430.107041	0.308096439	48.52239553	ATP-dependent zinc metalloprotease
Cluster-32430.135820	42.25128969	0	None
Cluster-32430.126776	0	61.17712304	Armadillo repeat-containing kinesin-like protein 1
Cluster-32430.71626	0	38.61313743	None
Cluster-32430.96634	37.23079965	0	None
Cluster-66662.1	58.81173229	0	None
Cluster-32430.83417	0	77.77617466	U-box domain-containing protein 44
Cluster-32430.192010	0	34.74414326	None
Cluster-32430.107551	23.65590037	192.7729235	Retrovirus-related Pol polyprotein from transposon TNT 1-94
Cluster-32430.132687	0	31.07604787	Alliin lyase
Cluster-32430.75749	31.25506638	0	Retrovirus-related Pol polyprotein from transposon TNT 1-94
Cluster-32430.30318	0	30.49094931	Pentatricopeptide repeat-containing protein
Cluster-32430.139928	29.46271667	0	None
Cluster-32430.93908	39.08297361	0	None
Cluster-32430.124207	0	27.65046076	Protein PHYLLO, chloroplastic
Cluster-32430.76732	0	27.75531509	None
Cluster-32430.94502	27.76571514	0	None
Cluster-32430.4608	0	67.33953135	Beta-glucosidase 25
Cluster-32430.132255	0	26.45207389	None
Cluster-32430.64383	0	51.98272839	Transcription factor bHLH121
Cluster-32430.80726	15.54530596	92.97608668	OTU domain-containing protein
Cluster-32430.81837	32.37499427	0.608515183	OTU domain-containing protein
Cluster-32430.147003	86.34561334	0	None
Cluster-32430.86775	42.79196066	0.377786653	None
Cluster-32430.71391	1.232385756	35.41500125	Gibberellin 20 oxidase 2
Cluster-32430.76615	8.557363811	68.96448585	None
Cluster-32430.155576	25.13024238	0	Probable phospholipid-transporting ATPase 5
Cluster-32430.45689	26.64544957	0	None
Cluster-32430.90497	3.379414339	44.61029633	ABC transporter G family member 28
Cluster-32430.54290	111.4511526	0.304257591	Probable leucine-rich repeat receptor-like protein kinase
Cluster-32430.80743	28.97679345	134.5901905	None
Cluster-32430.26814	0	24.12823491	None

Cluster-32430.181365	222.8903063	27.49475682	Premnaspirodiene oxygenase
Cluster-32430.66296	10.47527892	74.57476166	None
Cluster-32430.77681	244.6628103	68.90960028	Trans-cinnamate 4-monooxygenase
Cluster-32430.182348	23.51444168	0	None
Cluster-32430.80765	130.4146582	30.66156694	Receptor-like protein kinase BRI1-like 3
Cluster-32430.167555	23.17176142	0	RING domain-containing protein 2
Cluster-32430.61256	53.34746574	5.469630021	None
Cluster-32430.147617	0	26.32247068	Auxin response factor 23
Cluster-32430.116955	0.308096439	40.61288753	Sodium/hydrogen exchanger 2
Cluster-32430.124946	22.65475002	0	None
Cluster-32430.95809	34.20384633	0	None
Cluster-32430.69004	28.16953199	0.608515183	Deoxynucleoside triphosphate triphosphohydrolase SAMHD1 homolog
Cluster-30209.1	0	22.25376551	None
Cluster-32430.187776	22.25331987	0	None
Cluster-32430.36768	0	21.42734725	None
Cluster-32430.72346	12.32385756	76.53617908	Leucine-rich repeat receptor-like protein kinase
Cluster-32430.103842	4.621446584	47.43273568	SNF2 domain-containing protein CLASSY 1
Cluster-32430.93247	26.10081104	0	Uncharacterized mitochondrial protein
Cluster-32430.114245	89.22539073	13.23894443	None
Cluster-32430.22715	0	24.02278629	None
Cluster-32430.149248	21.64549571	0	None
Cluster-32430.81481	6.399410971	51.8841443	Uncharacterized protein
Cluster-32430.118496	1.257491922	48.8704969	None
Cluster-32430.179614	30.27690026	0	None
Cluster-32430.59832	0	20.7836349	None
Cluster-44018.0	0	20.69041744	None
Cluster-32430.163095	0	20.84597771	None
Cluster-32430.144527	0	20.60406292	None
Cluster-27356.0	0	20.53157877	None
Cluster-32430.163964	0	46.46372469	None
Cluster-32430.36115	76.87501498	13.56184551	LINE-1 retrotransposable element ORF2 protein
Cluster-32430.124571	26.79121349	110.201541	Protein RADIALIS-like 5
Cluster-32430.149181	49.9394496	2.43406073	None
Cluster-32430.147993	0	19.68592273	None
Cluster-32430.119078	625.5650297	1613.708747	6.4 kDa proline-rich protein
Cluster-32430.125552	19.98563354	0	Retrovirus-related Pol polyprotein from transposon TNT 1-94
Cluster-32430.41247	0	19.52126602	None
Cluster-32430.59195	20.35580667	0	None
Cluster-32430.74146	5.047645236	58.70339815	Pentatricopeptide repeat-containing protein

Cluster-32430.105335	110.556808	26.25117511	None
Cluster-32430.140355	0	18.90424869	None
Cluster-32430.185285	31.76387789	2.102063209	Polyadenylate-binding protein 1
Cluster-32430.79704	193.1201302	42.02559293	UPF0481 protein
Cluster-32430.178547	36.24443365	3.400079873	None
Cluster-32430.168371	500.4407479	181.9179282	Cytochrome P450 84A1
Cluster-41012.1	0	19.06622129	None
Cluster-32430.199547	57.42867496	5.780894234	None
Cluster-32430.141273	48.73677458	5.780894234	E3 ubiquitin-protein ligase
Cluster-32430.187970	18.72357162	0	None
Cluster-32430.110011	72165.29051	15263.4717	RNA replication protein
Cluster-32430.110201	4416.3668	10728.67305	Osmotin-like protein
Cluster-32430.71737	0	37.01504201	None
Cluster-37193.0	0	18.44920396	None
Cluster-29862.0	18.69103683	0	Phosphoinositide phospholipase C 2
Cluster-32430.201933	28.84255623	0	None
Cluster-30209.2	18.48464283	0	None
Cluster-32430.167775	18.43916932	0	None
Cluster-32430.110907	0.924289317	2213.893168	RNA replication protein
Cluster-32430.143893	0	20.44522505	Fanconi anemia group D2 protein homolog
Cluster-32430.145506	53.07869196	7.656408545	None
Cluster-32430.188659	29.06619418	0	None
Cluster-66303.0	34.90816216	2.812892291	Calreticulin
Cluster-32430.174186	1.232385756	26.62195514	None
Cluster-32430.52972	19.09135805	85.7170103	None
Cluster-32430.181484	6.329303223	47.20737457	DNA repair protein XRCC2 homolog
Cluster-32430.1018	28.1851605	0.304257591	None
Cluster-32430.41607	18.03441237	82.03414253	None
Cluster-32430.30225	17.94709525	0	None
Cluster-32430.90492	35.61876311	3.777866526	ABC transporter G family member 28
Cluster-32430.115147	234.5853279	78.47819465	Protein FAM214B
Cluster-32430.111587	0	42.40204732	Phototropin-2
Cluster-32430.87832	49.24289631	8.017190112	Protein trichome birefringence-like 3
Cluster-32430.116589	49.22757089	158.9023207	Scarecrow-like protein 3
Cluster-32430.110475	867344.2706	94345.10504	RNA replication protein
Cluster-32430.102725	45.54449585	6.683976278	Myosin-12
Cluster-32430.123770	42.6926795	0.912772774	None
Cluster-32430.124484	22.92906848	92.90837645	None
Cluster-32430.142231	41.33614036	4.563863869	Phospholipase D delta
Cluster-32430.108883	1.278968195	5520.159918	RNA replication protein
Cluster-32430.118311	186.8979399	50.50006047	None
Cluster-32430.201934	17.09449454	0	None
Cluster-32430.181457	17.0479121	0	None

Cluster-32430.179674	0	16.72492741	None
Cluster-32430.138137	4.114047095	49.36491684	LINE-1 retrotransposable element ORF2 protein
Cluster-32430.108836	6.858178985	16378.4296	RNA replication protein
Cluster-32430.135972	0	30.09496978	Putative E3 ubiquitin-protein ligase LIN-1
Cluster-32430.182902	37.3550873	4.322993993	Pentatricopeptide repeat-containing protein
Cluster-32430.40997	0	16.33968352	None
Cluster-41012.0	16.84134926	0	None
Cluster-32430.92390	8.448535848	49.22845148	Cytochrome c biogenesis CcmF C-terminal-like mitochondrial protein
Cluster-32430.109922	0.924289317	1219.682155	Uncharacterized ORF4 protein
Cluster-32430.130078	35.08723213	3.458694458	Pentatricopeptide repeat-containing protein
Cluster-32430.161717	16.76603076	0	None
Cluster-2331.2	20.79511287	0	None
Cluster-32430.12585	0	16.04288316	None
Cluster-32430.48385	0	15.93206712	Plastidal glycolate/glycerate translocator 1
Cluster-54685.2	0	15.95071061	None
Cluster-32430.134404	803.4489562	338.9334031	Cytosolic sulfotransferase 17
Cluster-32430.87908	0	15.84839671	None
Cluster-58617.2	15.91722878	0	Pentatricopeptide repeat-containing protein
Cluster-32430.132754	71.17991985	15.3187788	1,4-alpha-glucan-branching enzyme 3
Cluster-32430.187390	30.66888733	2.471347715	Major sperm protein isoform beta
Cluster-32430.202065	17.38854333	0	None
Cluster-32430.197702	32.02061849	1.51114661	None
Cluster-32430.121303	0	25.35913485	Protein PHYLLO, chloroplastic
Cluster-32430.68939	17.90286494	0	None
Cluster-37193.1	15.54107363	0	None
Cluster-32430.77837	24.26661773	0	None
Cluster-32430.183150	55.95403361	11.33359958	ATP-dependent DNA helicase pif1
Cluster-32430.49325	11.00933086	56.48902338	None
Cluster-32430.127388	0	14.99423852	Uncharacterized mitochondrial protein
Cluster-32430.109798	0.308096439	1156.016297	RNA replication protein
Cluster-32430.17630	1.2491232	22.89420026	None
Cluster-32430.62985	0	21.04464379	None
Cluster-32430.190012	8.643437735	49.39847561	Beta-glucosidase 31
Cluster-32430.110626	27.9508017	97.41794825	Cyclin-dependent kinase G-2
Cluster-20050.0	0	14.76142017	None
Cluster-32430.76189	20.32609593	0.755573305	None
Cluster-32430.108837	0.316465161	478.0593421	RNA replication protein
Cluster-32430.121	15.1217971	0	None
Cluster-32430.161747	15.13601359	0	None
Cluster-32430.86339	0.632930322	22.03153913	None
Cluster-32430.59769	14.97731095	0	None

Table S2 Primers used in this study

Gene symbol	Forward primer (5' to 3')	Reverse primer (5' to 3')	Purpose
AsGA20ox	ATGGACTCCATTTTTCTCCCC	TCACATATTTCCAGTTCTTTTAATC	CDS clone
AsGA20ox	TATCAAGTGATGGAAAGCTACAA	AGAGAAACGGTCGCTATGTG	RT-qPCR
AsINV	ATGTCTTCGTTTAGAGACTTGGAG	CTAGAGATCCATTAGGTTACTGTTTG	CDS clone
AsINV	TCTCATGCTTCTGACGCTTG	ATACGCACCCGCTTTCAAC	RT-qPCR
AsCYP735	GTTCTAACTGGCAGTATAGTCGA	TGATCATCTCAGTGTCCGTCA	RT-qPCR
AsAHK	TGGAAGGAGGAAAGATGTCA	GATCTGTTAAACCAGGCAAAC	RT-qPCR
AsAUX	GAGAAGCATTGGAGGTTTAGAAT	CTTTAGCAATGGGTCGATCA	RT-qPCR
AsBGLU31	GTATTTGCTGTTACCTGTGGCT	TCCAAAGCTGCTCCTTCTAC	RT-qPCR
ACT	CAGGAGTTATGGTTGGAATGG	AGCACGGGATGTTCTTCA	RT-qPCR
